# Aetiological agents of cerebrospinal meningitis: a retrospective study from a teaching hospital in Ghana

**DOI:** 10.1186/1476-0711-11-28

**Published:** 2012-10-04

**Authors:** Michael Owusu, Samuel Blay Nguah, Yaw Agyekum Boaitey, Ernest Badu-Boateng, Abdul-Raman Abubakr, Robert Awuley Lartey, Yaw Adu-Sarkodie

**Affiliations:** 1 Kumasi Centre for Collaborative Research in Tropical Medicine, Kumasi, Ghana; 2 Department of Child Health, Komfo Anokye Teaching Hospital, Kumasi, Ghana; 3 Department of Microbiology, Komfo Anokye Teaching Hospital, Kumasi, Ghana; 4 Department of Clinical Microbiology, Kwame Nkrumah University of Science and Technology, Kumasi, Ghana

**Keywords:** Meningitis, *Streptococcus pneumoniae*, *Cryptococcus neoformans*, Ghana

## Abstract

**Abstracts:**

**Background**

*Meningitis* is an important cause of morbidity and mortality in low-resource settings. In sub-Saharan Africa, the meningitis belt has been characterized by particularly high and seasonal incidences of bacterial meningitis extending throughout life. Despite the progress being made in treating the condition, the mortality rates continue to be high, ranging between 2% and 30% globally. In Ghana, the mortality rate of meningitis has been estimated to range from 36% to 50%. However little information is available on the pathogens contributing to meningitis and their antimicrobial susceptibilities. Updated information is essential to adjust the recommendations for empirical treatment or prevention of meningitis which could have immense implications for local and global health.

**Methods**

We retrospectively reviewed laboratory records of all patients suspected of bacterial meningitis who underwent a lumbar puncture from January 1, 2008 to December 31, 2010. Data were retrieved from laboratory record books and double entered into a Microsoft® excel spreadsheet.

**Results**

Records of 4,955 cerebrospinal fluid samples were analysed. Of these, 163 (3.3%, 95%CI: 2.8% to 3.8%) were confirmed meningitis and 106 (2.1%, 95%CI: 1.7% to 2.6%) were probable meningitis cases. Confirmed meningitis cases were made up of 117 (71.8%) culture positive bacteria, 19 (11.7%) culture positive *Cryptococcus neoformans* and 27(16.6%) Gram positive bacteria with negative culture. The most prevalent bacteria was *Streptococcus pneumoniae* 91 (77.7%), followed by *E.coli* 4 (3.4%), *Salmonella species* 4 (3.4%), *Neisseria meningitidis* 3 (2.5%), *Pseudomonas species* 3(2.5%) and others. Pneumococcal isolates susceptibility to penicillin, chloramphenicol and ceftriaxone were 98.9% (95%CI: 94.0% to 100.0%), 83.0% (95%CI: 73.4% to 90.1%) and 100.0% (95%CI: 95.8% to 100.0%) respectively.

**Conclusion**

*Streptococcus pneumoniae* is an important cause of meningitis among all age groups and its susceptibility to penicillin and ceftriaxone still remains very high. Ghanaians of all ages and possibly other developing countries in the meningitis belt could benefit from the use of the pneumococcal vaccine. Other bacterial and fungal pathogens should also be considered in the management of patients presenting with meningitis.

## Background

Cerebrospinal meningitis (CSM) is a major cause of morbidity and mortality in many parts of the world [1-3]. Despite the progress being made in treating the condition, the mortality rates continue to be high, ranging between 2% and 30% globally [4-6]. In Ghana, the mortality rate of meningitis has been estimated to range from 36% to 50% [7,8].

Apart from epidemics, at least 1.2 million cases of meningitis are estimated to occur with estimated annual deaths of 170,000 [9-11]**.** Complications such as epilepsy, mental retardation, deafness and other related neurological defects are observed in 10% to 20% of those who survive [12-14]. The estimated median risk of at least one major or minor sequel from bacterial meningitis after discharge from the hospital is 19.9% (range 12.3–35.3%) [14]. In middle and low-income countries, acute bacterial meningitis remains the fourth leading cause of disability [14]. The prevalence of bacterial meningitis in these countries is higher compared to developed countries.

Bacterial meningitis is caused by a number of organisms but beyond the neonatal period, over 90% of infections are caused by *Streptococcus pneumoniae (S. pneumoniae)*, *Haemophilus influenza (H. influenza)* and *Neisseria meningitidis (N. meningitidis)*[14]*.* Over the last two decades however, the causative agents of meningitis has changed with the introduction of new highly effective vaccines [15]. Haemophilus influenzae type b (Hib) used to be a common cause of bacterial meningitis worldwide before the Hib vaccines [16]. However more recently, *S. pneumoniae* and *N. meningitidis* have become the major organisms causing meningitis. In countries with high HIV prevalence, *Cryptococcus neoformans* may also be significant.

In many African countries including Ghana which lie within the meningitis belt, epidemic cases of acute bacterial meningitis caused by different subtypes of *N. meningitidis* and *S. pneumoniae* have been reported [17-23]. However most of the data were collected during outbreaks as such the pathogens detected were skewed to *S. pneumoniae* and *N. meningitidis*. Information on pathogens contributing to meningitis in hospital based studies is limited. Two hospital based studies in Ghana reported *S. pneumoniae*, *N. meningitidis and H. influenza* as pathogens associated with meningitis [7,24]. However these studies were only conducted among few populations of infants and children. Furthermore the contributions of other bacterial and fungal agents were not indicated.

Information from laboratory based surveillance is important in determining the most common aetiology of meningitis pathogens. It is further necessary for improving the clinical management of cases, guiding therapeutic decisions and for designing preventive strategies. In Ghana for instance, the only vaccine given to protect children at birth from meningitis is *Haemophilus influenza type b* vaccine. Vaccines to protect individuals against *N. meningitidis* are only given during outbreaks and pneumococcal vaccines are yet to be introduced.

This study is therefore important as it provides information on meningitis causing pathogens circulating throughout the years and also justifies the importance of vaccines or other control measures as a strategy for improving population health. The aim of this study was therefore to find out the different pathogens responsible for meningitis and their antibiotic susceptibility patterns.

## Methods

### Study area

The study was carried out at the Komfo Anokye Teaching Hospital (KATH), the second largest tertiary medical facility in Ghana. KATH is approximately a one thousand bed tertiary medical facility located in Kumasi, the capital of Ashanti region with a total projected population of 4,839,100. Kumasi lies in the central forest belt of Ghana, situated at 6.72^0^N 1.60^0^W, approximately 290 m above sea level and approximately 200km inland from Atlantic coast. The teaching hospital apart from taking care of patients located in the Ashanti region also attends to referral cases from the northern, western and eastern parts of Ghana. Its catchment population has been estimated to be 10 million. The hospital attends to an average of 400,000 out-patient cases and 41,000 in-patients a year [25]. The mortality rate as estimated in 2009 was 8.66% with meningitis ranked as one of the top ten causes of death [25].

The Ashanti region where the hospital is located has a tropical climate with two rainy seasons, April—June and September—November. During the period from December to March, the harmattan (a dry wind carrying dust from the Sahara desert) blows across Ghana from the northeast. Kumasi and the south of the country are most affected by the harmattan in January and early February.

### Study design and data collection

We retrospectively reviewed laboratory records of all patients suspected of bacterial meningitis who underwent lumbar puncture from January 1, 2008 to December 31, 2010. Data were retrieved from laboratory record books and double entered unto Microsoft® excel spreadsheet. Demographic data, clinical diagnoses, isolated organisms, cerebrospinal fluid (CSF) appearance and antibiotic susceptibility profile of organisms were collected. Six patients with bloody CSF appearance with no organism isolated were excluded from the data. Similarly any sample received from a patient who had a second sample submitted within 3 weeks of the first sample was counted as a single case. All coagulase negative staphylococci with cell counts less than 10 cells/mm^3^ were regarded as contaminants.

### Case definitions

Cases collected from the laboratory records were classified into probable and confirmed meningitis. Probable bacterial meningitis was defined as a case with no bacteria identified in the CSF but with leukocytosis of greater than or equal to 100 white blood cell/mm^3^ with greater than 60% being neutrophils [26-28]. A confirmed case of meningitis was defined as one of the following: Bacterial or cryptococcal isolation from CSF culture or a positive Gram Stain with no bacterial growth [26,27].

### CSF processing and bacterial or fungal culture

All CSF samples were processed at the bacteriology unit of KATH using standard microbiology techniques. Standard CSF analysis includes physical evaluation (divided into clear, hazy, cloudy, turbid, bloody and xanthochromic), WBC count per ml (determined manually with a modified Neubauer counting chamber) and differential white cell count.

Microbiological analysis included direct wet mount, Gram staining and India ink staining of the CSF deposits and culturing on chocolate agar, blood agar and sabouraud agar. All culture plates suspected of bacterial and fungal organisms were incubated at 37^0^C for 24–48 hours in 5% carbon dioxide environment and room temperature respectively. Any colonies observed were further processed and pathogens identified by standard microbiological techniques adapted from the WHO laboratory manual for diagnosing bacterial meningitis [29]. *N. meningitidis* species were identified by Gram stain, Oxidase test and colony morphology while *H. influenza* identification was based on X and V growth factors. Optochin sensitivity for confirmation of *S. pneumoniae* was based on a zone size of 14mm or more. Fungal growths on sabouraud agar were confirmed using indian ink and analytical profile index (bioMerieux, France).

All other bacteria isolates were identified using conventional biochemical methods including urease and indole production, citrate utilization, hydrogen sulphide gas production and fermentation of sugars. The biochemical media used included Simon’s Citrate medium, Urea and Triple Sugar Iron agar (TSI).

### Antimicrobial susceptibility testing

Antimicrobial susceptibility testing was done for all bacterial isolates using the Kirby-Bauer disk diffusion method. The antibiotics used were ceftriaxone (30μg), chloramphenicol (30μg), cefotaxime (30μg), oxacillin (1μg), amikacin (30μg), gentamicin (10μg), and septrin (23.75/1.25μg) (Oxoid, UK).

Susceptibility testing was performed by inoculating cultures directly in 0.85% saline and adjusted to a turbidity of 0.5 McFarland using densitometer. Inocula were poured on Nutrient Agar for non-fastidious organisms and Chocolate agar for fastidious organisms. Plates were read after overnight incubation and zone diameters were interpreted according to zone recommendation from CLSI. All isolates showing intermediate susceptibilities were classified as sensitive. Penicillin resistance was based on an oxacillin zone size of less than 19mm.

For all testing, control organisms received from the world health organisation external quality control support were included.

#### Ethical approval

The study protocol was approved by the Committee for Human Research, Publications and Ethics (CHRPE) of KATH and School of Medical Sciences, KNUST, Kumasi, Ashanti region, Ghana.

#### Statistical analysis

Data was exported to Stata SE version 11.2 for analysis. The ages were stratified as follows: less than 1 month, 1 to < 5 years, 5 to < 18 years, 18 to < 50 years and greater than or equal to 50 years. Proportions for the various organisms and demographic features were expressed as percentages with or without their binomial exact 95% confidence intervals.

## Results

Over the three year period of 2008-2010, records of 4,955 CSF samples of suspected cases of meningitis were analysed. Of these, 163 (3.3% 95%CI: 2.8% to 3.8%) were confirmed meningitis and 106 (2.1%, 95%CI: 1.7% to 2.6%) were probable meningitis cases. The numbers of confirmed meningitis cases were 64 in 2008, 41 in 2009 and 31 in 2010 whereas probable meningitis occurred in 75, 52 and 36 infected patients for the same periods respectively (Table 1). Confirmed meningitis cases were made up of 117 (71.8%) culture positive bacteria, 19 (11.7%) culture positive *Cryptococcus neoformans* and 27 (16.6%) Gram positive bacteria with negative culture. There was a general reduction in the number of meningitis cases over the three year period. The most prevalent bacteria was *S. pneumoniae* 91 (77.7%), followed by *E.coli* 4 (3.4%) or *Salmonella species* 4 (3.4%), *N. meningitidis* 3 (2.5%) or *Pseudomonas species* 3 (2.5%), *Klebsiella species* 2 (1.7%) and *Enterobacter species* 1 (0.9%) or *H. influenza* 1(0.9%). The majority of those infected with *S. pneumoniae* 53 (58.2%) were below 18 years of age and the highest cases of *Cryptococcus neoformans* 17(89.4%) occurred among patients between 18 and 50 years. Other Gram negative rods such as *Salmonella species*, *Pseudomonas species*, *E.coli* and *H. influenza* were more common among children less than 5 years compared to older children and adults (Table 2). This difference was however not significant.

**Table 1:** Yearly distribution of meningitis pathogens

**Pathogens**	**Year n (%)**			
	**2008**	**2009**	**2010**	**Total**
*N.meningitidis*	0	2 (4.9)	1 (3.2)	3 (2.2)
*S.pneumoniae*	53 (82.8)	22 (53.7)	16 (51.6)	91 (66.9)
*H.influenzae*	0	0	1(3.2)	1 (0.7)
*Cryptococcus neoformans*	5 (7.8)	6 (14.6)	8 (25.8)	19 (14.0)
*Pseudomonas species*	0	0	1 (3.2)	1 (0.7)
*Pseudomonas aeruginosa*	0	0	2 (6.5)	2 (1.5)
*E.coli*	2 (3.1)	2 (4.9)	0	4 (2.9)
*Klebsiella species*	1 (1.6)	0	1 (3.2)	2 (1.5)
*Staphylococcus aureus*	0	3 (7.3)	0	3 (2.2)
*Salmonella species*	2 (3.1)	2 (4.9)	0	4 (2.9)
*Enterobacter species*	0	1 (2.4)	0	1 (0.7)
Coliforms	0	2 (4.9)	0	2 (1.5)
Coagulase negative staphylococcus.	1 (1.6)	1 (2.4)	1 (3.2)	3 (2.2)
Total	64 (100.0)	41 (100.0)	31 (100.0)	136 (100.0)

**Table 2:** Meningitis pathogens and age group distribution

	**Age grouping (years) n(%)**				
**Pathogens**	**≤ 1mth**	**1mth to <5yrs**	**5yrs to <18yrs**	**18yrs to <50yrs**	**>= 50yrs**
*N.meningitidis.*	1(6.7)	0	1(3.4)	1(2.0)	0
*S.pneumoniae*	8(53.3)	21(70.0)	24(82.8)	30(60.0)	8 (66.7)
*H.influenzae*	0	1(3.3)	0	0	0
*Salmonella species*	1(6.7)	3(10.0)	0	0	0
*Pseudomonas spp*.	0	1(3.3)	0	0	0
*Pseudomonas aeruginosa*	2(13.3)	0	0	0	0
*E.coli*	1(6.7)	1(3.3)	0	1 (2.0)	1(8.3)
*Cryptococcus neoformans.*	0	0	2(6.9)	15 (30.0)	2 (16.7)
*Staphylococcus aureus*.	0	3(10.0)	0	0	0
*Klebsiella species.*	0	0	0	1 (2.0)	1 (8.3)
*Enterobacter species*	1(6.7)	0	0	0	0
Coliforms	1(6.8)	0	0	1 (2.0)	0
Coagulase negative staphylococcus	0	0	2(6.9)	1 (2.0)	0
Total	15 (100.0)	30 (100.0)	29 (100.0)	50 (100.0)	12 (100.0)

The median cell count of patients infected with *S. pneumoniae* was 362.5 (55-1247) cells/mm^3^ and that of *N. meningitidis* was 12700 (3,200-14000) cells/mm^3^. Results of antimicrobial susceptibility testing *of S. pneumoniae and N. meningitidis* isolates with penicillin, ceftriaxone and cefotaxime shows almost all isolates were susceptible (Table 3). Only twenty five percent of *Salmonella spp* isolates were susceptible to chloramphenicol.

**Table 3:** Antimicrobial susceptibility patterns of meningitis pathogens

	**Percentage Sensitivity %(n/N)**						
**Antibiotic**	***Streptococcuspneumoniae***	***Neisseriameningitidis***	***Haemophilusinfluenzae***	***Salmonellaspecies***	***Pseudomonasspecies***	***E. coli***	**Others**^**b**^
Penicillin	98.9 (89/90)	100.0 (3/3)	0(0/1)	---	--	--	57.1 (4/7)
Chloramphenicol	83.0 (73/88)	100.0 (2/2)	100.0 (1/1)	25.0 (1/4)	--	66.7 (2/3)	77.8 (7/9)
Ceftriaxone	100.0 (86/86)	100.0 (2/2)	100.0 (1/1)	100.0 (3/3)	100.0 (2/2)	100.0 (3/3)	83.3 (5/6)
Cefotaxime	100.0 (16/16)	100.0 (2/2)	--	100.0 (3/3)	--	66.7 (2/3)	50.0 (2/4)
Septrin	--	--	--	0 (0/4)	--	0 (0/3)	14.3 (1/7)
Ampicillin	--	--	0(0/1)	0 (0/3)	--	0 (0/4)	14.3 (1/7)
Gentamycin	--	--	--	100 (2/2)	50 (1/2)	50.0 (1/2)	60.0 (3/5)
Ceftazidime	--	--	--	0 (0/1)	0 (0/2)	--	--
Amikacin	--	--	--	100.0 (2/2)	50 (1/2)	100 (3/3)	100.0 (3/3)

Others^b:^ This represents the cumulative percentage of the following organisms: *Klebsiella species, Enterobacter species, Staphylococcus aureus*, Coagulase negative staphylococcus and Coliforms. *Pseudomonas species* includes *Pseudomonas aeruginosa* organisms. Antibiotics were not tested against all organisms with dash (--).

**Figure 1:**
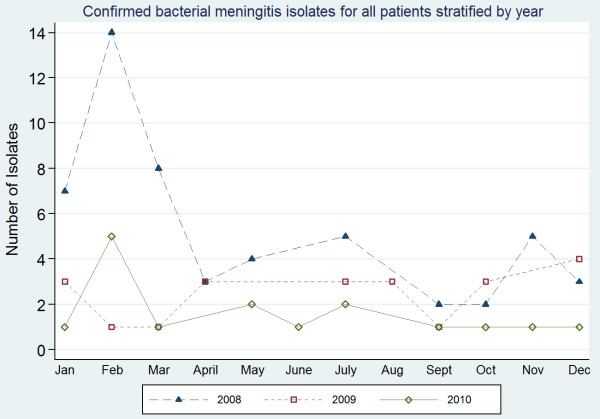
** Confirmed meningitis for all patients stratified by year.** The figure describes the monthly distribution of all patients classified as having confirmed meningitis.

Seasonal variation of confirmed meningitis cases was examined over the three year period (Figure 1). Confirmed meningitis cases were generally higher in the year 2008 compared to the other years. The highest peak occurred in February 2008. Lower monthly prevalence was however recorded in the year 2010 except for the month of February.

## Discussion

Cerebrospinal meningitis is a major cause of mortality and morbidity in both children and adults. In Ghana, cases of meningitis outbreaks are mostly reported in the northern part of the country and has been attributed to the low humidity in that area. In the years 1996-1997, the three northern regions of Ghana for instance recorded 18,703 cases of meningitis out of which 1,356 lost their lives during an outbreak. [30,31]. Similar cases of meningitis have been reported in other parts of Ghana [7,24,32] however hospital based information on the different aetiological agents, antimicrobial susceptibility patterns and the seasonality is limited.

In our study the prevalence of confirmed and probable meningitis were 3.3% and 2.1% respectively. Our result is similar to other surveillance (based on cultures) of meningitis in other parts of Africa [33,34]. The prevalence however appears low compared to some reports from the northern part of Ghana. This is because our reports were from all patients suspected of meningitis while reports from northern Ghana were mostly obtained during outbreaks. We however believe the prevalence could have been higher if techniques using polymerase chain reactions were applied in testing the samples [35,36]. Another reason that could contribute to the low meningitis prevalence is the possible use of antibiotics before hospital admission, a common practice in many developing countries [35,37]. Even though we have no records on the number of patients on antibiotics before admission, our observation show that most of the admitted cases referred from other primary healthcare facilities had been managed with antibiotics before referral. Future studies are however needed to confirm this.

The most prevalent bacteria was *S. pneumoniae* occurring mostly among patients less than 18 years of age. Neonates accounted for 8.7% and children from one month to less than 5 years accounted for 23%. Our result is similar to previous studies done by Holliman *et al.*, [8] where they found majority of *S. pneumoniae* infections occurred in children and young patients. Adult pneumococcal meningitis which accounted for 41.8% of infections was equally significantly identified as reported in other studies [38]. A possible reason for the predominant prevalence of pneumococcal meningitis could be as a result of auto-infection from colonizing bacteria in the nasopharynx. Previous studies have shown the prevalence of nasopharyngeal colonization with streptococcal pneumoniae to be 51.4% [39] in Ghana. The auto-transmission from the nasopharynx into the meninges is therefore highly possible especially during the dry season when cracks and injuries tend to occur in the nasopharynx. Countries that introduced pneumococcal conjugate vaccines have however reported a reduction in the cases of pneumococcal meningitis [40].

We found one case of *H. influenzae* over the three year period. This low prevalence is quite remarkable and could be explained by the introduction of Hib vaccines in Ghana in 2002. The impact of Hib vaccine on the reduction of meningitis has been reported by Renner et al [15]. Other developing countries such as Turkey have also reported low Hib meningitis prevalence [41]. It is however possible that the infected child may not have been vaccinated against *H. influenzae*. Though the Hib immunization status of the child with the Hib meningitis could not be determined it is possible he might have been born in a rural area where access to healthcare and vaccination may be difficult. It is also possible that the under reporting of meningitis cases might have contributed to the low numbers. This is because some developed countries like the USA which introduced vaccines against Hib meningitis decades ago still reports prevalence of 6.7% [42].

The contribution of other bacteria pathogens to meningitis apart from *N. meningitidis, S. pneumoniae and Haemophilus influenzae* has not been widely reported in many developing countries. The present study identified *E.coli, Salmonella species, Pseudomonas species, Klebsiella species, Staphylococcus aureus and Enterobacter species* as contributing to 12% of all bacterial isolates among children and infants. Some studies in developing countries have similarly reported these observations [43-45]. Contrary to these findings however, children and neonates in developed countries have been reported to have predominance of Group B streptococcus and *Lysteria monocytogenes*[46]. The reasons for the non-identification of *Lysteria monocytogenes* in our laboratory could be due to less attention given to the laboratory diagnosis of this pathogen. This is because *Lysteria monocytogenes* resembles diphtheroids and could easily be regarded as contaminants. Furthermore reagents such as esculin and hippurate hydrolysis or CAMP test (Christie, Atkins and Munch-Peterson) used for confirming *Lysteria monocytogenes* are not readily available in our laboratory.

Of interest in our study is the identification of *Cryptococcus neoformans,* occurring mostly in patients between the ages of 18 and 50 years. Previous studies in Ghana (Kumasi) did not identify *Cryptococcus neoformans* in meningitis patients perhaps due to the small number of study patients [32]. The identification of *Cryptococcus neoformans* is however not surprising since the number of people living with HIV/AIDS continue to increase even though the national HIV prevalence had reduced from 1.8% in 2008 to 1.5% in 2010. The contribution of *Cryptococcus neoformans* to meningitis has similarly been reported to mostly occur in HIV endemic African countries [47-49].

All isolates tested with ceftriaxone were 100% susceptible with the exception of *Staphylococcus aureus*. Eighty three percent (83%) of *S. pneumoniae* isolates were susceptible to chloramphenicol whereas 98.9% were susceptible to penicillin. Our study found no evidence of high level resistance against chloramphenicol and penicillin. Previous studies in Ghana have however documented pneumococcal penicillin resistance rates varying from 8%-31% [7,8,50] and chloramphenicol resistance rates of 5-20.6% [50]. The difference in the resistance patterns could be due to the methodology used. The sensitivity patterns of our isolates were done using only the Kirby-Bauer method which could underestimate the resistance levels. On the other hand, the susceptibility of *S. pneumoniae, N. meningitidis and H. influenzae* to ceftriaxone has been mostly reported to be 100% [24,39,50].

Appropriate treatment for meningitis depends on the local antimicrobial susceptibility patterns. In Ghana, the hospital antibiotic policy recommends penicillin and chloramphenicol as the first choice of meningitis treatment and ceftriaxone is considered as alternative [51]. This study therefore emphasizes the need for clinicians to continuously rely on ceftriaxone as the best choice of drug for the treatment of meningitis.

Seasonal distribution of confirmed meningitis cases were noted to increase from December through January and peaked in February of 2008 (figure 1). The next highest peak of confirmed meningitis also occurred in February of 2010. This trend is not surprising as the dry season reaches its peak during these months and thus provides the conditions for destruction of the mucosal defences thus making individuals more susceptible to meningitis [22,52]. These conditions may however not entirely promote meningitis since the trend in 2009 was quite different. More studies on seasonal variation is therefore needed to understand the exact seasons that significantly promote meningitis.

## Conclusion

*S. pneumoniae* still remains a major cause of bacterial meningitis among all age groups and its susceptibility to penicillin, chloramphenicol and ceftriaxone still remains high. Ghanaians of all ages and possibly other developing countries in the meningitis belt could benefit from the use of the pneumococcal vaccine. The contribution of other pathogens such as *E.coli*, *Klebsiella species*, *Salmonella species* and *Cryptococcus neoformans* in developing countries cannot be underestimated in the treatment and management of meningitis. This study emphasizes the need for extensive hospital based surveillance of meningitis causing pathogens in developing countries.

## Abbreviations

CSM: Cerebrospinal Meningitis; HIV: Human immunodeficiency virus; CSF: Cerebrospinal fluid; KATH: Komfo Anokye Teaching Hospital; WBC: White blood cells; TSI: Triple Sugar Iron; CHRPE: Committee for Human Research, Publication and Ethics; CLSI: Clinical and Laboratory Standards Institute; *S. pneumoniae*: *Streptococcus pneumoniae*; *H. influenzae*: *Haemophilus influenzae*; *N. meningitidis*: *Neisseria meningitidis*.

## Competing interests

Authors have no competing interests.

## Authors' contributions

YAB, BBE, AAR and ALR co-worked on data collection and organisation. SBN performed statistical analysis of the data and contributed to writing and interpretation of the manuscript. O.M designed, initiated the study and wrote the manuscript. YAS contributed to the design and writing of the manuscript. All authors have read and approved the manuscript.
